# Supplementary material for: FlowDock: Geometric flow matching for generative protein–ligand docking and affinity prediction
Source: Bioinformatics. 2025 Jul 15;41(Suppl 1):i198–206. doi: 10.1093/bioinformatics/btaf187 (PMC12261468; doi:10.1093/bioinformatics/btaf187)
Supplement: btaf187_Supplementary_Data [file btaf187_supplementary_data.zip › Morehead.17 Alt Text File.pdf]

Main Text:

Figure 1 (Morehead.17.fig.1.pdf): "An overview of biomolecular distribution modeling with FlowDock."

Figure 2 (Morehead.17.fig.2.pdf): "Protein-ligand docking success rates of each baseline method on the PoseBusters Benchmark set (n=308). Error bars: 3 runs."

Figure 3 (Morehead.17.fig.3.pdf): "Comparison of each flexible docking method's protein conformational changes made for the PoseBusters Benchmark set."

Figure 4 (Morehead.17.fig.4.pdf): "Protein-ligand docking success rates of each baseline method on the DockGen-E set (n=14). Error bars: 3 runs."

Figure 5 (Morehead.17.fig.5.pdf): "Comparison of each flexible docking method's protein conformational changes made for the DockGen-E set."

Figure 6 (Morehead.17.fig.6.pdf): "Comparison of DynamicBind and FlowDock's predicted structures (w/o hydrogens) and crystal PDBBind test example 6I67."

Figure 7 (Morehead.17.fig.7.pdf): "Protein-ligand binding affinity prediction rankings for the CASP16 ligand prediction category (n=140)."

Table 1: "The average structure prediction runtime (in seconds) and peak memory usage (in GB) of baseline methods on a 25% subset of the Astex Diverse dataset using an NVIDIA 80GB A100 GPU for benchmarking. The symbol '-' denotes a result that could not be estimated."

Table 2: "Binding affinity estimation using PDBBind test set."

Supplementary Materials:

Algorithm 1: "Training pseudocode for FlowDock."

Algorithm 2: "Inference pseudocode for FlowDock."

Figure 1 (Morehead.17.sup.1.pdf): "Comparison of FlowDock's predicted structure states (w/o hydrogens) for CASP16 superligand pose pharma target L3008."

Figure 2 (Morehead.17.sup.2.pdf): "Comparison of the protein-ligand structure prediction results of FlowDock and the deep learning ensembling method MULTICOM_ligand in terms of their binding pocket-aligned ligand RMSDs for the CASP16 superligand pose pharma targets (n=301)."

Figure 3 (Morehead.17.sup.3.pdf): "Comparison of the protein-(multi-)ligand structure prediction results of FlowDock and the deep learning ensembling method MULTICOM_ligand in terms of their binding pocket-aligned ligand RMSDs for the CASP16 superligand pose pharma targets (n=126)."

Figure 4 (Morehead.17.sup.4.pdf): "Analysis of the protein-ligand structure prediction results of FlowDock in terms of its binding pocket-aligned ligand RMSDs for the chemically dissimilar (multi-)ligand PoseBusters Benchmark targets (n=18)."
