# Supplementary material for: FlowDock: Geometric flow matching for generative protein–ligand docking and affinity prediction
Source: Bioinformatics. 2025 Jul 15;41(Suppl 1):i198–206. doi: 10.1093/bioinformatics/btaf187 (PMC12261468; doi:10.1093/bioinformatics/btaf187)
Supplement: btaf187_Supplementary_Data [file btaf187_supplementary_data.zip › Morehead.17.sup.6.pdf]

Journal Title Here, 2022, 1–7
doi: DOI HERE
Advance Access Publication Date: Day Month Year
Paper

Supplementary Materials for: "FlowDock: Geometric Flow Matching for Generative Protein-Ligand Docking and Affinity Prediction"

Alex Morehead[1,*] and Jianlin Cheng[1]

[1]Department of Electrical Engineering & Computer Science, NextGen Precision Health, University of Missouri-Columbia, W1024 Lafferre Hall, 65211, Missouri, USA

*Corresponding author. acmwhb@missouri.edu

FOR PUBLISHER ONLY Received on Date Month Year; revised on Date Month Year; accepted on Date Month Year

A. Geometric flow matching training and inference

We characterize FlowDock's training and sampling procedures in Sections 3.5 (Training) and 3.6 (Sampling) of the main text, respectively. To further illustrate how training and inference with FlowDock work, in Algorithms 1 and 2 we provide the corresponding pseudocode. For more details, please see our accompanying source code at `https://github.com/BioinfoMachineLearning/FlowDock`.

Algorithm 1 Training

Require: Training examples of binding site-aligned apo (holo) protein (ligand) structures, protein sequences, ligand SMILES strings, and binding affinities $\{(X^P_{a_i}, X^P_{h_i}, X^L_{h_i}, S_i, M_i, B_i)\}$

for all $(X^P_{a_i}, X^P_{h_i}, X^L_{h_i}, S_i, M_i, B_i)$ **do**
  Extract $x^P_1, x^L_1 \leftarrow \text{HeavyAtoms}(X^P_{h_i}, X^L_{h_i})$
  Sample $x^P_0 \leftarrow \text{ESMFold}(S_i) + \epsilon, \quad \epsilon \sim \mathcal{N}(0, \sigma = 1e^{-4})$
  Sample $x^L_0 \leftarrow \text{HarmonicPrior}(M_{i_{frag}}), \quad \forall frag \in M_i$
  Sample $t \sim \mathcal{U}(0, 1)$
  Concatenate $x_0 = \text{Concat}(x^P_0, x^L_0)$
  Concatenate $x_1 = \text{Concat}(x^P_1, x^L_1)$
  Interpolate $x_t \leftarrow t \cdot x_1 + (1-t) \cdot x_0$
  Predict $\hat{X}_{h_i} \leftarrow \text{NeuralPLexer}(S_i, M_i, x_t, t)$
  Predict $\hat{B}_i \leftarrow \text{ESDM}_{aff}(S_i, M_i, \text{StopGrad}(\hat{X}_{h_i}))$
  Optimize losses $\mathcal{L}_X := \lambda_X \cdot \text{FAPE}(X_{h_i}, \hat{X}_{h_i}) + \mathcal{L}_B := \lambda_B \cdot \text{MSE}(\hat{B}_i, B_i), \quad \lambda_X = 0.2, \quad \lambda_B = 0.1$
end for

Algorithm 2 Inference

Require: Protein sequences and ligand SMILES strings (S, M)
Ensure: Sampled top-5 heavy-atom structures $\hat{X}$ with confidence scores $\hat{C}$ and binding affinities $\hat{B}$

Sample $x^P_0 \leftarrow \text{ESMFold}(S) + \epsilon, \quad \epsilon \sim \mathcal{N}(0, \sigma = 1e^{-4})$
Sample $x^L_0 \leftarrow \text{HarmonicPrior}(M_{frag}), \quad \forall frag \in M$
Concatenate $x_0 = \text{Concat}(x^P_0, x^L_0)$
for $n \leftarrow 0$ to i **do**
  Let $t \leftarrow \frac{n}{i}$ and $s \leftarrow \frac{n+1}{i}$
  Predict $\hat{X} \leftarrow \text{NeuralPLexer}(S, M, x_n, t)$
  if $n = i - 1$ **then**
    Predict $\hat{C} \leftarrow \text{ESDM}_{conf}(S, M, \hat{X})$ # Pre-trained
    Predict $\hat{B} \leftarrow \text{ESDM}_{aff}(S, M, \hat{X})$
    Rank top-5 $\hat{X}$ and $\hat{B}$ using $\hat{C}$
    return $\hat{X}, \hat{C}, \hat{B}$
  end if
  Extract $\hat{x}_1 \leftarrow \text{HeavyAtoms}(\hat{X})$
  Align $x_n \leftarrow \text{RMSDAlign}(x_n, \hat{x}_1)$
  Interpolate $x_{n+1} = clamp(\frac{1-s}{1-t} \cdot \eta) \cdot x_n + clamp((1 - \frac{1-s}{1-t}) \cdot \eta) \cdot \hat{x}_1, \quad \eta = 1$
end for

© The Author 2022. Published by Oxford University Press. All rights reserved. For permissions, please e-mail: journals.permissions@oup.com

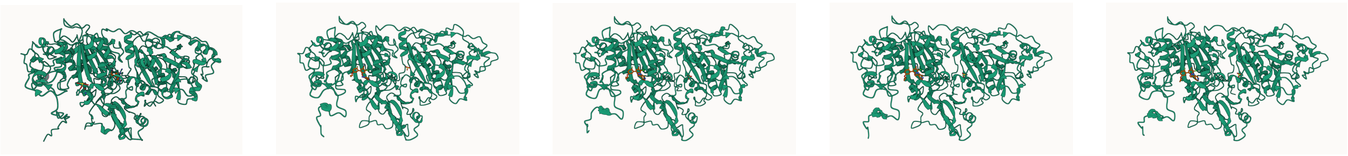

FlowDock Init. (t=1/40) | **FlowDock Interm.** (t=10/40) | **FlowDock Interm.** (t=20/40) | **FlowDock Interm.** (t=30/40) | **FlowDock Final** (t=40/40)

Fig. 1: Comparison of FlowDock's predicted structure states (w/o hydrogens) for CASP16 superligand pose pharma target L3008.

B. Structure generation example trajectory

To illustrate one of FlowDock's interpretable structure generation trajectories using conditional flow matching, in Figure 1, we report FlowDock's predicted structural states for CASP16 superligand pose pharma target L3008, notably a *multi*-ligand pose target, in evenly spaced increments throughout FlowDock's generation trajectory. In short, we see that FlowDock enables multi-ligand protein complexes to be predicted through concise flow trajectories, yielding early protein and ligand conformational changes following the model's initial binding pocket prediction.

C. CASP16 structure prediction results

In Figure 2, we compare the protein-ligand structure prediction RMSDs of FlowDock and MULTICOM_ligand (Morehead et al., 2025), a top-5 multi-model deep learning prediction method in the CASP16 ligand prediction category, for the 231 superligand pose pharma targets made available during the 16th Critical Assessment of Techniques for Structure Prediction (CASP16). As these results demonstrate, FlowDock, as a standalone deep learning method, achieves competitive structure predictions for many of the new CASP16 ligand targets. Similarly, Figure 3 illustrates that FlowDock and MULTICOM_ligand are approximately tied in terms of their ability to structurally model CASP16's 56 *multi*-ligand protein complexes, further highlighting the broad applicability of FlowDock's structure predictions in diverse drug discovery settings.

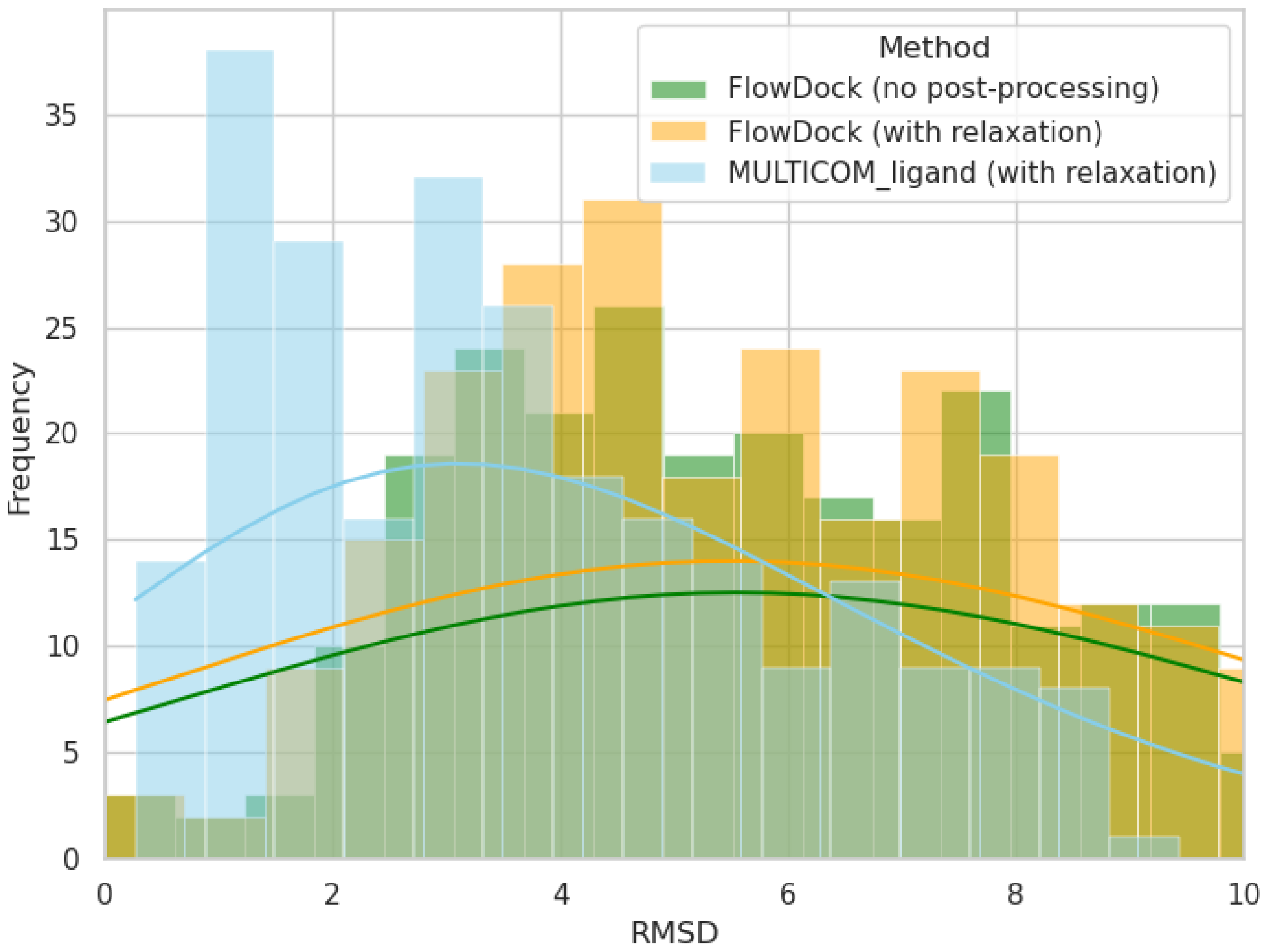

Fig. 2: Comparison of the protein-ligand structure prediction results of FlowDock and the deep learning ensembling method MULTICOM_ligand in terms of their binding pocket-aligned ligand RMSDs for the CASP16 superligand pose pharma targets (n=301).

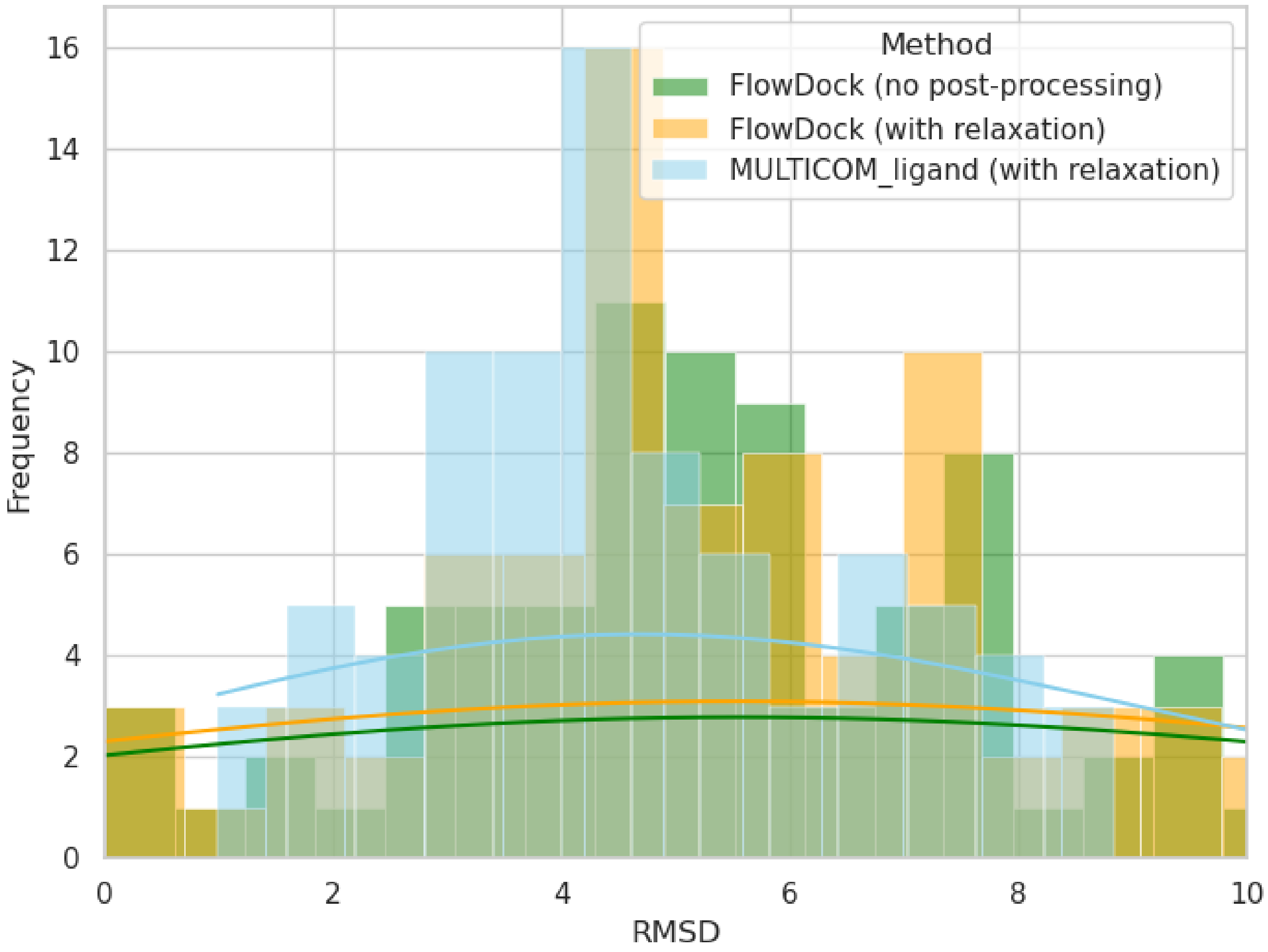

Fig. 3: Comparison of the protein-(multi-)ligand structure prediction results of FlowDock and the deep learning ensembling method MULTICOM_ligand in terms of their binding pocket-aligned ligand RMSDs for the CASP16 superligand pose pharma targets (n=126).

D. PoseBusters Benchmark ligand dissimilarity structure prediction results

To investigate FlowDock's chemical generalization capabilities, in Figure 4, we illustrate the structure prediction performance of FlowDock for chemically dissimilar (Tanimoto similarity < 0.6) ligands associated with the same protein target in the PoseBusters Benchmark dataset. Figure 4 shows that FlowDock's average ligand RMSD of each of these (multi-)ligand protein targets is approximately 2 Å, with a standard deviation around 1 Å, highlighting that its predictions for chemically dissimilar intra-protein ligands are of high average accuracy and demonstrate generalizability with the consistency of FlowDock's average inter-ligand RMSD differences.

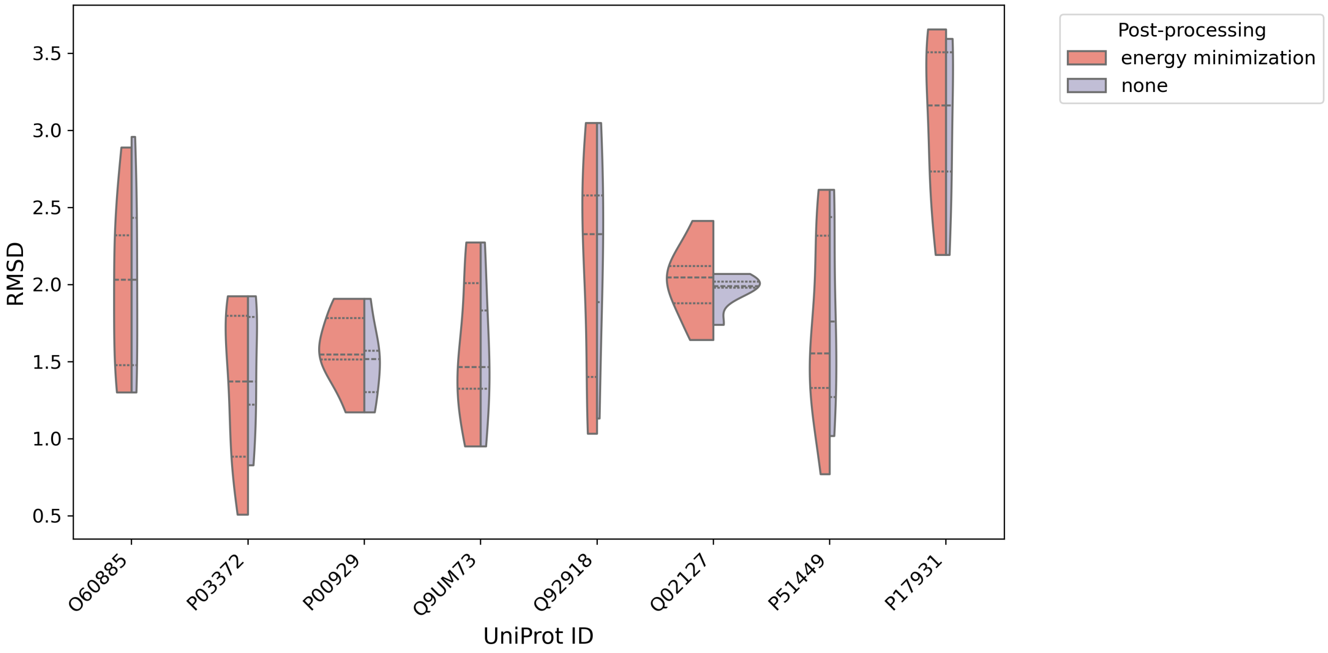

Fig. 4: Analysis of the protein-ligand structure prediction results of FlowDock in terms of its binding pocket-aligned ligand RMSDs for the chemically dissimilar (multi-)ligand PoseBusters Benchmark targets (n=18).

References

A. Morehead, J. Liu, P. Neupane, N. Giri, and J. Cheng. Protein-ligand structure and affinity prediction in casp16 using a geometric deep learning ensemble and flow matching. *Authorea*, 2025.
