# Supplementary figures and images for: FlowDock: Geometric flow matching for generative protein–ligand docking and affinity prediction
Source: Bioinformatics. 2025 Jul 15;41(Suppl 1):i198–206. doi: 10.1093/bioinformatics/btaf187 (PMC12261468; doi:10.1093/bioinformatics/btaf187)

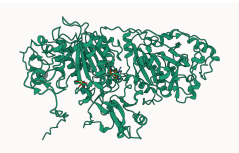

**FlowDock Init.**  
(t=1/40)

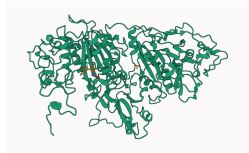

**FlowDock Interm.**  
(t=10/40)

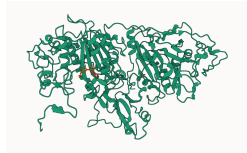

**FlowDock Interm.**  
(t=20/40)

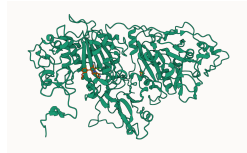

**FlowDock Interm.**  
(t=30/40)

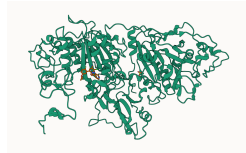

**FlowDock Final**  
(t=40/40)

Supplement: btaf187_Supplementary_Data [file btaf187_supplementary_data.zip › Morehead.17.sup.1.pdf]

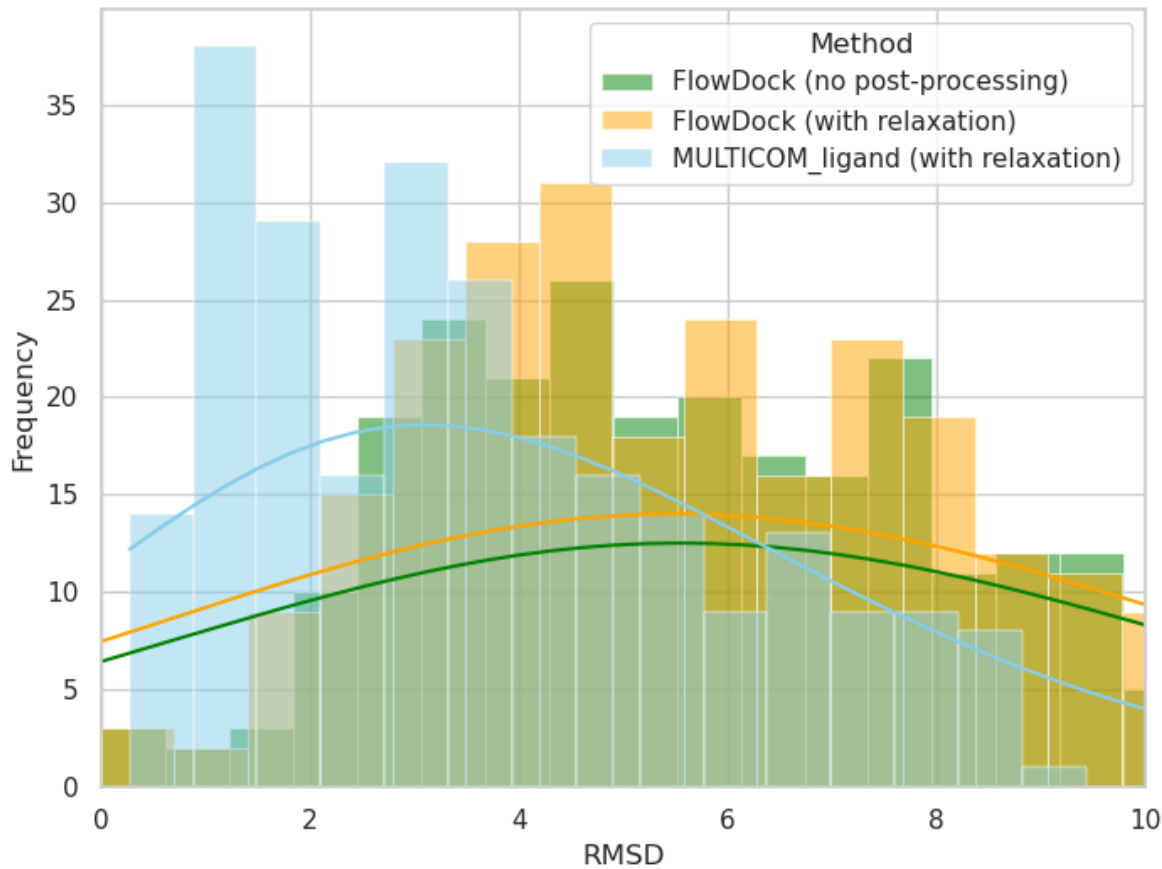

Supplement: btaf187_Supplementary_Data [file btaf187_supplementary_data.zip › Morehead.17.sup.2.pdf]

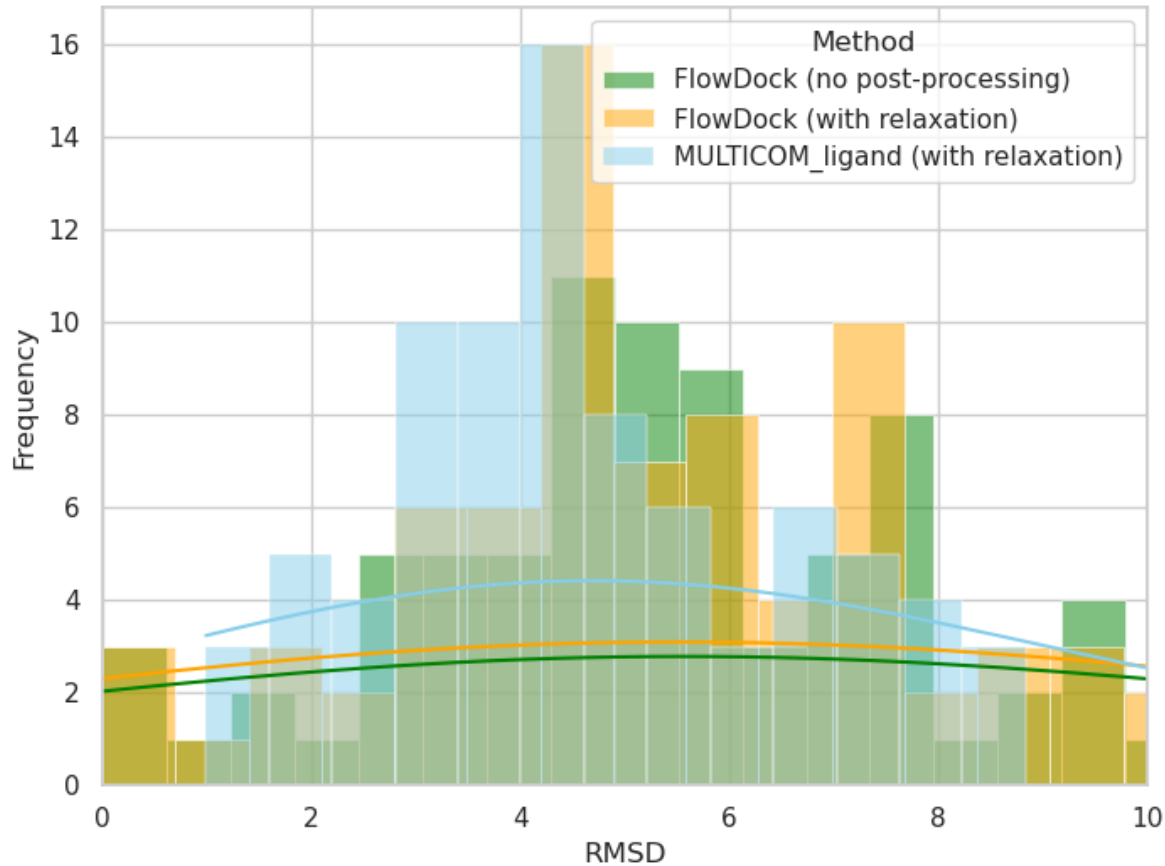

Supplement: btaf187_Supplementary_Data [file btaf187_supplementary_data.zip › Morehead.17.sup.3.pdf]

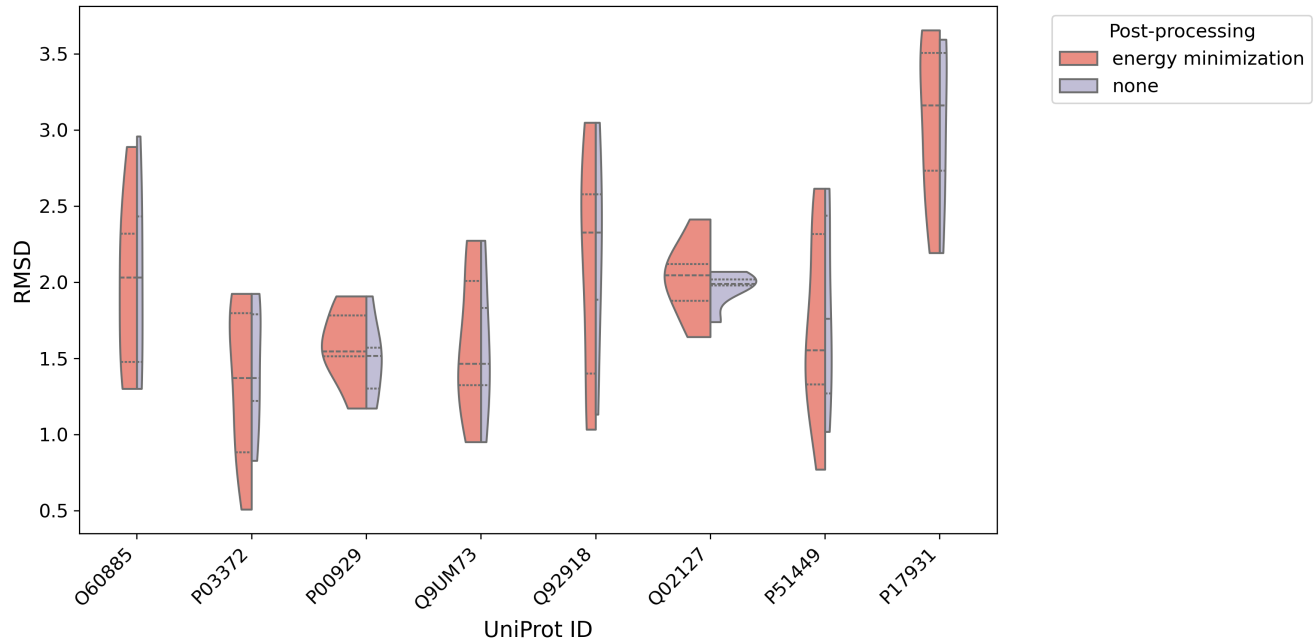

Supplement: btaf187_Supplementary_Data [file btaf187_supplementary_data.zip › Morehead.17.sup.4.pdf]
